# The Role of Angiographic Imaging in the Treatment of Spinal Vascular Malformations

**DOI:** 10.3390/medsci13040266

**Published:** 2025-11-13

**Authors:** Camilla Giulia Calastra, Ada Ayechu Abendaño, Raluca-Ana-Maria Barna, Federica Orellana, Simone Baffelli, Ameet Aiyangar, Annapaola Parrilli

**Affiliations:** 1 Center for X-Ray Analytics, Empa—Swiss Federal Laboratories for Materials Science and Technology, Überlandstrasse 129, 8600 Dübendorf, Switzerlandfederica.orellana97@gmail.com (F.O.); 2 Mechanical Systems Engineering, Empa—Swiss Federal Laboratories for Materials Science and Technology, Überlandstrasse 129, 8600 Dübendorf, Switzerland; 3 Faculty of Medicine, University of Bern, Hochschulstrasse 6, 3012 Bern, Switzerland; 4 Spine Biomechanics, Balgrist University Hospital, Lengghalde 5, 8008 Zürich, Switzerland; 5 Scientific IT, Empa—Swiss Federal Laboratories for Materials Science and Technology, Überlandstrasse 129, 8600 Dübendorf, Switzerland; 6 Department of Diagnostic, Interventional and Pediatric Radiology (DIPR), Inselspital, Bern University Hospital, University of Bern, Rosenbühlgasse 27, 3010 Bern, Switzerland

**Keywords:** angiography, spinal vascular malformations, arteriovenous malformations, arteriovenous fistulas, digital subtraction angiography, indocyanine green videoangiography, MR angiography, CT angiography, treatment

## Abstract

Spinal vascular malformations (SVMs) are rare and heterogeneous lesions that may lead to progressive neurological decline or hemorrhage, posing significant challenges for management due to their complex angioarchitecture and proximity to critical neural structures. This review examines the role of angiographic imaging modalities used intraoperatively and postoperatively in guiding treatment, confirming therapeutic success, and informing follow-up strategies. We summarize evidence on two-dimensional digital subtraction angiography (2D DSA), indocyanine green videoangiography (ICG–VAG), and emerging adjunctive techniques. 2D DSA remains the reference standard, offering superior temporal and spatial resolution for real-time visualization of vascular anatomy, catheter navigation, and embolic delivery, though its invasive nature, radiation exposure, and two-dimensional projection limit long-term applicability. ICG–VAG provides a complementary, non-ionizing method for intraoperative fluorescence imaging, aiding in shunt localization and venous preservation, although its restricted field of view and limited capacity for quantitative analysis reduce its standalone value. Advances in quantitative angiographic metrics, patient-specific hemodynamic modeling, and artificial intelligence-driven image analysis are anticipated to enhance diagnostic accuracy and reproducibility. The development of standardized multimodal protocols will be crucial for optimizing patient-centered treatment of these complex and rare lesions.

## 1. Introduction

Spinal vascular malformations (SVMs) represent a heterogeneous group of vascular anomalies [1,2,3,4,5] that may be either congenital or acquired [6,7] and can be found at various levels of the spinal cord and its surrounding structures. The occurrence rate of SVMs is 3–16% of all space-occupying spinal lesions [8,9] and 5–9% of all central nervous system vascular malformations [8]. These lesions are broadly classified into arteriovenous fistulas (AVFs), which involve direct artery-to-vein connections (approximately 20% of SVMs), and arteriovenous malformations (AVMs), which feature a nidus—an intervening tangle of abnormal vessels without capillary interposition—accounting for the remaining 80% [8]. Among the various classification systems proposed, the angioarchitectural scheme by Takai [10] remains a reference standard. It delineates five major types of spinal arteriovenous shunts: dural AVFs (Type I), intramedullary glomus AVMs (Type II), intramedullary juvenile AVMs (Type III), perimedullary AVFs (Type IV), and extradural AVFs (Type V).

SVMs have significant clinical implications, as they can cause progressive neurological decline through venous hypertension, ischemia, or hemorrhage. Patients may present with nonspecific symptoms such as back pain, radiculopathy, or sensory disturbances, while more advanced disease can manifest with spastic paraparesis, sphincter dysfunction, or acute myelopathy [11,12]. Hemorrhagic events are particularly associated with intramedullary AVMs (Types II and III), carrying a substantial risk of sudden neurological deterioration [10]. Treatment depends on lesion type and angioarchitecture. For example, dural AVFs (Type I) are often curable by microsurgical disconnection of the draining vein or endovascular embolization, both of which are associated with favorable outcomes [13,14]. Conversely, intramedullary AVMs are among the most challenging lesions to treat due to their complex vascular networks and high-flow dynamics, and their management often requires a multidisciplinary approach combining surgery, embolization, and in selected cases, radiotherapy. Despite advances in microsurgical and endovascular techniques, treatment carries non-negligible risks of morbidity and recurrence, underscoring the importance of accurate diagnosis and tailored therapeutic strategies.

Managing SVMs is highly challenging due to their intricate angioarchitecture, variable clinical presentation, and frequent association with critical neural structures [2]. Accurate delineation of these lesions requires high-resolution imaging that can capture subtle anatomical details for optimal endovascular treatment planning. During endovascular treatment of spinal AVMs, intraoperative 2D Digital Subtraction Angiography (2D DSA) remains the gold standard for real-time guidance. Its high temporal and spatial resolution allows precise superselective catheterization, identification of the shunt zone, and monitoring of embolic agent progression, enabling immediate detection of residual arteriovenous transit or dangerous reflux into non-target vessels.

In microsurgical management, indocyanine green videoangiography (ICG–VAG) has emerged as a valuable adjunct, offering high-resolution real-time visualization of vascular anatomy without radiation exposure. Administered intravenously, ICG–VAG enables clear identification of the fistulous point and arterialized draining veins, providing immediate confirmation of successful disconnection by demonstrating the absence of early venous filling. Its use has been shown to improve surgical outcomes and reduce the need for postoperative 2D DSA, while also aiding preservation of adjacent normal venous structures.

In general, achieving optimal surgical or endovascular outcomes requires strategies that minimize perioperative complications, reduce procedure-related morbidity, and ensure complete lesion obliteration. Therefore, the continuous refinement of imaging and intraoperative navigation techniques is essential to improving long-term neurological outcomes and optimizing patient follow-up. Despite the evolution of both surgical and endovascular techniques for treating SVMs, there is no standardized approach to guide intraoperative angiographic strategy. Most available data derive from small, retrospective series focused on specific lesion subtypes or single imaging modalities. This narrative review aims to synthesize the current evidence on the intraoperative and postoperative role of angiographic techniques in treating SVMs, emphasizing their respective strengths and limitations, and highlighting emerging innovations that may refine therapeutic decision-making.

## 2. Literature Selection

This review was designed as a narrative synthesis rather than a systematic review. Nevertheless, a structured literature search was conducted to ensure comprehensive and unbiased coverage of the topic. Relevant literature was identified through PubMed, Cochrane, Embase, and Web of Knowledge using combinations of terms including *spinal vascular malformations*, *arteriovenous fistula*, *arteriovenous malformation*, *intraoperative angiography*, *digital subtraction angiography*, and *indocyanine green videoangiography*. The search focused on studies published at any time up to August 2025. Additional references were identified through citation tracking and expert knowledge of the field, with emphasis placed on clinical relevance, imaging innovations, and intraoperative applications.

## 3. Intraoperative Role of 2D DSA

First introduced in the early 1970s and widely adopted by the late 1970s and 1980s, 2D DSA revolutionized vascular imaging by replacing film-based subtraction with electronic processing, enabling real-time removal of background structures such as bone while enhancing visualization of contrast-filled vessels [15]. The fundamentals of 2D DSA were first elucidated at the University of Wisconsin, and by 1980, the first commercial units were being sold. Initially developed for diagnostic cerebrovascular imaging, its application rapidly expanded to peripheral and spinal angiography as advances in fluoroscopic technology, catheter design, and flat-panel detector systems improved resolution and acquisition speed. Reports of intraoperative use of 2D DSA first appeared in the mid to late 1980s [16,17,18], marking the integration of 2D DSA into surgical workflows.

**Figure 1:**
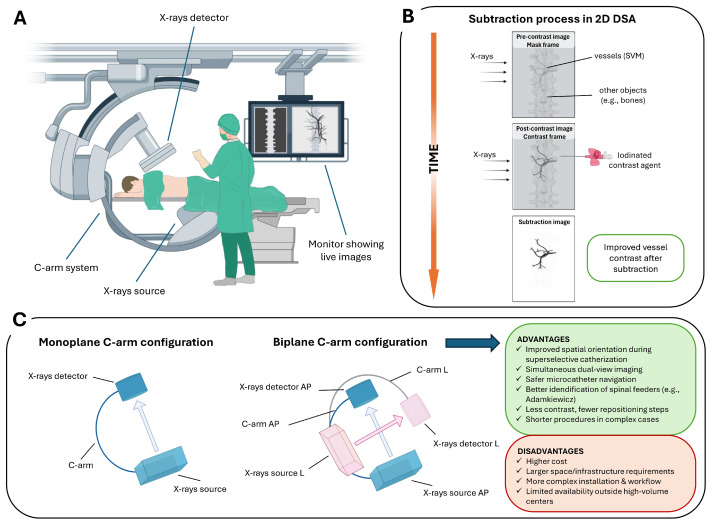
2D DSA workflow and C-arm configurations for SVMs: (**A**) C-arm angiography system used for spinal procedures, including an X-ray source, detector, and real-time fluoroscopic imaging display. (**B**) Schematic of the subtraction process in 2D DSA. A pre-contrast mask frame is acquired, followed by a contrast-enhanced frame; digital subtraction suppresses background structures (e.g., bone), enhancing visualization of spinal vascular malformations. (**C**) Monoplane versus biplane C-arm configurations. Biplane systems provide simultaneous orthogonal projections (anteroposterior, AP, and lateral, L), improving catheter navigation and anatomical orientation in complex SVMs, at the cost of increased system complexity and infrastructure requirements.

Intraoperative 2D DSA is an advanced fluoroscopic technique that provides real-time dynamic imaging of the vascular system, used during a wide spectrum of endovascular interventions (Figure 1A). The fundamental principle involves the acquisition of a baseline “mask” image prior to contrast administration, followed by intra-arterial injection of an iodinated contrast agent via a selectively positioned microcatheter within the spinal vasculature [19]. The post-contrast image is then digitally subtracted from the pre-contrast mask, effectively suppressing background anatomical structures and enhancing the visualization of vascular details (Figure 1B).

Several subtraction techniques have been developed to optimize image quality in different clinical settings. Temporal subtraction, the most widely used, compares pre- and post-contrast images to highlight dynamic vascular changes over time [20]. Dual-energy subtraction involves acquiring images at different X-ray energies to selectively suppress signals from bone or soft tissue, thereby enhancing the visibility of vascular structures [21]. Spectral or energy-based subtraction techniques can further isolate contrast-filled vessels from surrounding anatomy [22]. Hybrid subtraction, which integrates temporal and energy-based methods, is particularly effective in reducing motion artifacts and improving overall image clarity [23].

The technique is performed using monoplane or biplane C-arm systems. Biplane systems offer simultaneous acquisition of orthogonal projections (e.g., anteroposterior and lateral), facilitating spatial orientation and compensating for the intrinsic limitations of 2D imaging in complex spinal vascular anatomy. Its high temporal and spatial resolution enables precise visualization of catheter navigation, accurate localization of the shunt zone during superselective catheterization, the visualization of normal structures that may be at risk during the treatment of these lesions (e.g., anterior spinal artery (ASA) or the artery of Adamkiewicz (AKA)) [24], and continuous monitoring of contrast dynamics. Compared to monoplane systems, biplane angiography provides simultaneous orthogonal projections, improving spatial orientation in the complex spinal vascular anatomy. This real-time dual-view capability facilitates safer and more precise superselective catheterization of segmental and radiculomedullary feeders, particularly when preserving spinal cord supply (e.g., the AKA) is critical (Figure 1C).

Real-time imaging may also reveal residual arteriovenous transit or abnormal venous drainage. This includes direct assessment of embolic agent progression and the ability to detect dangerous reflux into non-target vessels, as emphasized by Gemmete et al. in their technical overview of endovascular strategies for AVMs [25].

The most commonly used contrast agents in intraoperative 2D DSA are iodinated, water-soluble compounds, due to their high radiopacity and rapid intravascular distribution [26]. Among these, non-ionic, low-osmolar agents, such as Iohexol, Iodixanol, and Iopamidol, are preferred for their favorable safety profile, low incidence of adverse reactions, and effective vessel opacification [27]. These contrast media are typically injected intra-arterially via microcatheters selectively positioned in the spinal arterial feeders. Injection parameters, such as volume and flow rate, are tightly controlled, often via power injectors, to optimize imaging quality while avoiding contrast reflux or non-target embolization [15,28].

Various occlusive agents can be used to treat SVMs, either through percutaneous sclerotherapy or transarterial embolization. Sclerotherapy involves the direct injection of a sclerosing agent into the SVM under ultrasound or fluoroscopic guidance [29]. These agents damage the vascular endothelium, triggering inflammation, thrombosis, and eventually, fibrosis [29]. Over time, the fibrotic tissue may be reabsorbed, resulting in lesion reduction [30].

In transarterial embolization, embolic agents are delivered via microcatheters under angiographic control, allowing the operator to monitor progress in real time. Because most of these agents are radiopaque, the extent of embolization can be assessed after each injection. Among the most commonly used agents, N-butyl cyanoacrylate is a fast-acting adhesive mixed with an iodinated oil (e.g., Lipiodol), to enable fluoroscopic visualization [31,32]. Onyx, a tantalum-based embolic agent, offers a cohesive formulation that allows for slower and more controlled injection with the potential of deep nidus penetration [33]. Precipitating Hydrophobic Injectable Liquid is an iodine-based embolic agent with properties similar to Onyx, but it provides improved image homogeneity and fewer artifacts [34]. The choice of agent depends on lesion anatomy, flow characteristics, and operator preference.

In the context of SVMs, these advantages are particularly critical, as precise delineation of their intricate angioarchitecture and shunt points relies heavily on meticulous selective angiography. SVMs often feature small-caliber, tortuous arterial feeders originating from segmental vessels, necessitating careful catheterization and high-resolution angiographic imaging. Consequently, 2D DSA remains indispensable intraoperatively, not only to verify complete obliteration following microsurgical or endovascular intervention but also to identify residual shunts that may escape detection with other intraoperative imaging modalities.

While 2D DSA remains the gold standard for vascular imaging, it carries several important limitations in the evaluation and treatment of SVMs. Though modern systems mitigate risks, the technique is inherently invasive, with frequently prolonged procedures that may expose both patients and operators to significant radiation [35]. For example, 2D DSA often requires selective catheterization of all spinal cord feeding arteries, sometimes under general anaesthesia, and may involve staging to a second examination due to duration or patient motion. In complex embolization procedures, fluoroscopy may exceed 35 min, with a mean radiation dose of 347.1 Gy·cm^2^ and a diagnostic reference level as high as 482.7 Gy·cm^2^ for spinal dural AVFs embolization [36,37]. These levels are comparable to those reported for cerebral AVM embolizations, highlighting the complexity of these cases. Such exposure has been cited as a contraindication for embolization in specific populations, including pregnant patients, where alternative, non-ionizing methods like MR angiography are preferred.

Additionally, anatomical variability and lesion location further complicate spinal angiography. Lesions located in the cervical and upper thoracic regions are generally more accessible and better visualised due to shorter contrast travel distances and less overlapping vasculature. In contrast, lesions in the thoracolumbar and sacral regions often present technical challenges, such as limited catheter navigation, motion artifacts from respiration or bowel peristalsis, and reduced image clarity due to vascular crowding or contrast dilution [38]. Specifically, motion artifacts are a particularly significant limitation of 2D DSA [15]. Even subtle patient movement, physiological pulsation, or respiration can cause image misregistration, obscuring vascular detail and making it difficult to accurately assess vessel filling or embolic agent distribution during embolization. These artifacts may lead to false impressions of complete occlusion or cause operators to misinterpret flow dynamics in real time.

The two-dimensional nature of the technique is another key limitation. Planar 2D projection imaging can cause vessel overlap and obscure critical feeders, particularly in complex arterial territories. Identifying small feeding arteries, fistulous points, and their spatial relationships with adjacent osseous structures is fundamental in the treatment planning [39] and in the interventional procedure, to assess the depth of penetration of embolic agent or to evaluate residual nidus, especially in tortuous vasculature. Additionally, hemorrhagic complications, collateral branches, or residual flow, may also not be immediately evident in the absence of 3D prospective [39]. Suboptimal opacification, caused by factors such as intra-procedural hypotension, improper catheter positioning, or low contrast bolus delivery, can further compromise the evaluation process. This increases the risk of non-target embolization as the embolic material enters draining veins prematurely or refluxes into spinal cord-supplying vessels, potentially leading to severe neurological diseases. Although 2D DSA remains unmatched in depicting vascular morphology and flow dynamics, its use is largely limited to the assessment of vascular structures rather than surrounding tissues. Therefore, in the context of treating SVMs, its primary value lies in guiding and performing embolization, confirming treatment success, and supporting early post-treatment assessment, whereas late follow-up is typically performed using non-invasive imaging modalities.

## 4. Intraoperative Role of ICG–VAG

The potential of using ICG as a contrast agent in medical applications has been evident since the 1950s, at the time applied in the field of cardiology [40]. Its use extended rapidly in other medical fields, specifically ophthalmology [41], where the development of angiographic applications dates back to the 1970s [42,43]. In the mid-90s till early 2000, the technique became more widely adopted thanks to the technological development of digital imaging resolution [44].

ICG–VAG is an intraoperative fluorescence imaging technique widely used to visualize vasculature, assess blood flow, and evaluate tissue perfusion in real-time [45]. Unlike X-ray–based angiography, ICG–VAG does not require ionizing radiation or iodinated contrast media. Instead, it relies on ICG, a water-soluble near-infrared (NIR) fluorescent dye prepared as a sterile aqueous solution containing a small percentage of sodium iodide. Following intravenous (i.v.) injection, ICG rapidly binds to plasma proteins, remaining largely confined to the intravascular compartment, which makes vascular filling and circulation patterns highly visible.

**Figure 2:**
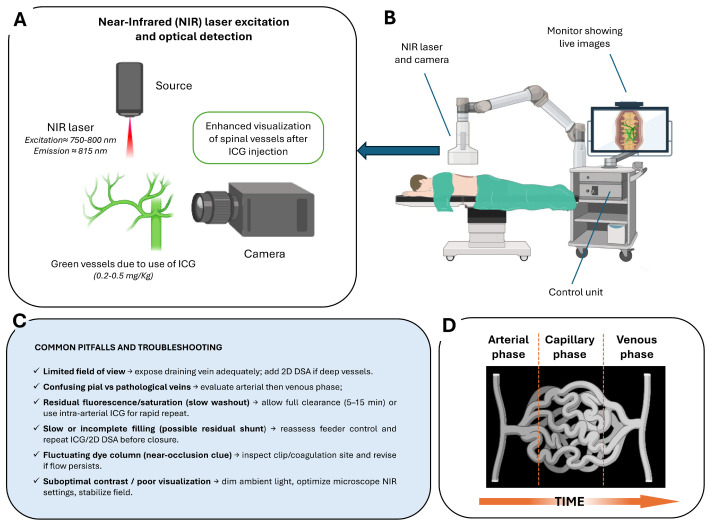
Intraoperative ICG videoangiography (ICG–VAG) workflow and principles: (**A**) NIR excitation and optical detection principle. Following intravenous administration of ICG, vessels are illuminated with NIR light, producing green fluorescence that allows real-time visualization of spinal vasculature. (**B**) Intraoperative setup with NIR laser/camera system and live image display integrated into the surgical microscope control unit. (**C**) Common pitfalls and troubleshooting strategies, including limited field of view, residual fluorescence, incomplete filling, near-occlusion signs, and optimization of contrast and visualization. (**D**) Schematic representation of the arterial, capillary, and venous phases over time following ICG injection.

The imaging process is based on tissue illumination with a specific excitation wavelength of approximately 750–800 nm, followed by detection of fluorescence emission at wavelengths above 800 nm [46] (Figure 2A). Modern operative microscopes are equipped with integrated NIR light sources, specialized optical filters, and high-sensitivity cameras to detect this emission and project fluorescence images directly onto the surgical field. The system allows for seamless switching between white-light microscopy and NIR fluorescence visualization without altering the surgical setup (Figure 2B,C).

After i.v. bolus administration (typically 0.2–0.5 mg/kg), ICG rapidly circulates through the arterial, capillary, and venous phases, producing a clear sequence of vascular filling visible within seconds (Figure 2D). In routine spinal vascular surgery, the i.v. route is standard, as it reliably demonstrates early venous filling and confirms complete disconnection following clip or coagulation. The washout time after i.v. injection typically ranges between 5 and 15 min, which is compatible with the surgical workflow in these lesions. However, in selected complex cases—such as perimedullary AVFs with multiple feeders (Type III/IV) or lesions in hybrid operating room settings—selective intra-arterial (i.a.) ICG injection may be preferred. The i.a. administration, delivered through a microcatheter positioned in the feeding segmental artery, produces a much sharper arterial-phase signal with minimal dilution and rapid washout (~1 min), allowing repeated injections to map individual feeders and distinguish true shunt points from bystanders. This technique is particularly valuable when the angioarchitecture is not clearly resolved with i.v. ICG alone, and when selective confirmation of flow obliteration is needed. Recent hybrid operating room (hybrid-OR) series have demonstrated precise feeder identification and faster intraoperative decision-making with the i.a. micro-boluses (~0.05 mg) [47,48,49,50].

In both approaches, ICG’s exclusive hepatic clearance permits repeated injection without significant risk of accumulation or systemic toxicity. ICG–VAG is generally safe, with a low incidence of adverse reactions and no nephrotoxicity, making it suitable even in patients with renal impairment [51].

In the context of SVMs, intraoperative ICG–VAG has become an increasingly valuable adjunct in surgical management, with its use reported across a growing number of patient series [52,53,54]. It provides immediate, high-resolution visualization of feeders, fistulous points, and venous drainage within the operative corridor. Its efficacy in accurately identifying arteriovenous shunting sites during microsurgical treatment has been demonstrated in comparative studies [51], where the use of fluorescence imaging significantly improved surgical outcomes compared with procedures performed without intraoperative angiographic guidance or with embolization alone. Precise localization of multiple arterial feeders using ICG–VAG eliminated the need for reoperation in the reported patient cohorts, underscoring its potential to reduce reliance on postoperative 2D DSA for confirmation of complete lesion obliteration.

Detailed vascular visualization is fundamental for preserving normal venous structure and preventing venous complications in rare conditions. ICG–VAG has been successfully employed for the microsurgical treatment of superior petrosal sinus dural AVFs [55]. In the procedure, ICG proved to be an efficient aid in preserving and distinguishing the co-existing normal superior petrosal vein adjacent to the fistula from the arterialized superior petrosal sinus.

Despite its advantages, the technique faces some limitations in spinal applications. One major constraint is that ICG fluorescence imaging is restricted to the surgical field and therefore cannot visualize vessels outside the microsurgical exposure. This makes its diagnostic capacity limited compared to preoperative or intraoperative 2D DSA, which can display the entire vascular territory [56]. Another limitation is related to the relatively low perfusion pressure. This factor can hinder the clear differentiation between arterial and venous phases in spinal dural AVFs, especially in cases with complex vascular architecture or small-caliber feeding arteries. To address this challenge, several technique refinements have been proposed. Efforts have been made to address this limitation and ad hoc methods have been implemented, such as pooling techniques for improving visualization of dural AVFs [57]. Such approaches have demonstrated improved signal intensity and image quality, illustrating the ongoing evolution of ICG–VAG protocols to optimize its performance in challenging spinal vascular cases. Additionally, ICG–VAG also provides only two-dimensional, surface-level visualization, as the fluorescent signal originates from vessels directly exposed under the microscope. Deeper or hidden vessels may not be visualized, which limits the ability of this method to fully delineate complex nidus structures or deep-seated arterial feeders [58]. Furthermore, ICG–VAG lacks the capability for quantitative flow analysis. While it is highly effective for confirming obliteration or flow direction qualitatively, it cannot provide detailed hemodynamic data comparable to that obtained from 2D DSA. Finally, repeated injections are feasible due to the dye’s safety profile, but each injection requires pausing the procedure for several seconds, which may slightly prolong operative time.

The simplicity, safety, and real-time feedback of ICG–VAG make it a valuable adjunct in the surgical management of SVMs, complementing endovascular strategies and aiding surgical decision-making regardless of prior embolization status. Ongoing technical refinements and its combination with other imaging modalities may further consolidate its role as a standard component of complex spinal vascular procedures.

To ensure conceptual continuity between SVM classification and intraoperative decision-making, Table 1 provides a structured algorithm linking lesion subtype, pathophysiology, angiographic modality, and operative goal.

**Table 1:** Intraoperative decision framework for SVMs. Summary of key pathophysiology, imaging strategy, surgical/endovascular rationale, and objective intraoperative endpoints for each spinal vascular malformation subtype, integrating 2D DSA, CE–trMRA, ICG–VAG, and neuromonitoring considerations.

SVM Subtype	Key Pathophysiology	Intraoperative Imaging Strategy	Operative Goal and Rationale	Objective Endpoint
**Dural AVF (Type I)**	Intradural venous reflux → venous hypertensive myelopathy; low-flow dural shunt at the nerve root sleeve	Full-spine 2D DSA (gold standard); adjunct CE–trMRA for pre-op level localization; optional feeder coil marking during diagnostic angiography for intra-op guidance	Definitive microsurgical disconnection of the intradural draining vein. Endovascular treatment (EVT) only in favourable anatomy (single feeder, direct shunt) when liquid embolic can penetrate the fistula and proximal outflow vein; avoid proximal-only coil/particulate embolization (high recurrence). Identify AKA; consider IONM	No early venous drainage on final 2D DSA and absent early ICG venous opacification; if EVT used, embolic cast extends into the proximal draining vein; preserved physiologic venous outflow [24,59]
**Perimedullary (Pial) AVF (Type IV)**	Direct pial artery–to–vein shunt; often high-flow; risk to ASA/PSA and cord perfusion; possible hemorrhage	2D DSA for feeder and ASA/PSA mapping; adjunct CE–trMRA for level targeting; mandatory ICG–VAG intra-op to verify closure and arterial patency	Eliminate pial shunt while preserving ASA/PSA supply. Low-flow: microsurgical disconnection. High-flow/multiple feeders: primary EVT with venous penetration; staged EVT + surgery if incomplete	No residual shunt with venous penetration; preserved ASA/PSA perfusion; no early venous filling on ICG and 2D DSA; angiographic cure at follow-up [60,61]
**Intramedullary Glomus AVM (Type II)**	Compact intramedullary nidus, often fed by ASA/PSA branches; may present with hemorrhage, progressive myelopathy, or rarely radiculopathy from prenidal aneurysm compression	High-resolution spinal 2D DSA mandatory; MRI/MRA to identify intramedullary flow voids and associated aneurysms; intra-op ICG useful only for superficial components	Goal is safe shunt and pressure reduction while preserving cord perfusion. Endovascular embolization preferred when feasible, especially for associated aneurysms; staged or partial embolization when ASA supply places cord at risk; microsurgical resection reserved for select cases with accessible components or incomplete EVT	Reduction or obliteration of nidus/aneurysm with preserved neurological function; complete angiographic cure when safely achievable; durable symptom relief and prevention of rebleeding [62,63]
**Juvenile / Diffuse AVM (Type III)**	Extensive multi-compartment nidus; aggressive hemodynamics	2D DSA for global flow pattern; ICG–VAG limited to exposed draining veins	Flow reduction and decompression rather than complete cure; staged EVT + surgery as needed	Controlled shunt volume; venous hypertension relieved; neurological stability
**Extradural AVF (Type V)**	Extradural arteriovenous shunt with epidural venous pouch; may exhibit intradural venous reflux causing venous hypertension	2D DSA to evaluate extradural pouch and confirm/exclude intradural reflux; adjunct intraoperative ICG–VAG if concern for intradural drainage	If intradural venous reflux: target disconnection of intradural draining vein and eliminate venous hypertension; if purely extradural: occlude epidural pouch and arterial feeders endovascularly, preserving spinal arterial supply	Complete occlusion of extradural shunt and epidural venous pouch with durable elimination of intradural reflux; intact spinal cord arterial perfusion; no residual shunt on final 2D DSA and no recurrence on follow-up MRI/MRA [64,65]

## 5. Intraoperative Role of Other Angiography Modalities

Beyond 2D DSA and ICG–VAG, other angiographic techniques have been explored intraoperatively to optimize surgical outcomes and SVMs’ management (Table 2). These methods, though less widely adopted, provide additional anatomical or functional information that can guide complex procedures and reduce the risk of incomplete treatment. Some studies addressed the surgical challenges posed by SVMs in which conventional microscopic approaches and standard ICG–VAG may fall short due to limited visualization. Ito et al. (2017) [66] pioneered the use of endoscope-integrated ICG–VAG to access and visualize both dural and perimedullary AVFs on the ventral cervical spinal cord. Such an approach allowed for dynamic assessment of blood flow in regions otherwise hidden from the microscope, expanding the field of view without requiring spinal cord rotation. Similarly, Mansour et al. (2019) [67] employed the PINPOINT endoscopic fluorescence imaging system to treat a ruptured ASA aneurysm associated with a craniocervical junction epidural AVF via a posterolateral approach. In both cases, the integration of endoscopic ICG–VAG enabled real-time visualization of the ventral spinal cord and facilitated safe clipping of the aneurysm while preserving the ASA, without the need for spinal cord traction or rotation.

Additionally, Fukuda et al. (2020) [68] introduced a novel combination of an anterior surgical approach via cervical corpectomy with angled endoscopy and fluorescein VAG (FL–VAG). By replacing ICG with fluorescein, which produces a brighter signal and works with smaller endoscopes, this technique enabled safe and complete obliteration of shunts in tight ventral spaces, marking a significant variant from traditional ICG-based methods. Similarly, Horiuchi et al. (2020) [69] used endoscope-integrated FL–VAG through a posterior suboccipital approach, demonstrating the ability of the technique to reveal complex vascular anatomy and real-time hemodynamics even in areas obscured by subarachnoid clots. FL–VAG allowed repeated intraoperative confirmation of residual flow and preserved critical vessels, enhancing the safety and precision of the procedure.

In a subsequent development, one study (Yu et al., 2024 [70]) implemented the “pressure cooker” technique for enhanced embolization control in epidural AVFs, representing a novel adaptation of the standard endovascular strategy. This method involves the use of two microcatheters: one positioned at the arteriovenous shunt for the delivery of a liquid embolic agent, and a second used to deploy coils proximally in the feeding artery to create a mechanical plug. This configuration prevents reflux of the embolic agent and facilitates its forward penetration into the fistulous point and draining veins. The main advantages of this technique are improved control over embolic agent distribution, reduced risk of non-target embolization, and increased likelihood of complete AVF obliteration even in complex anatomical locations.

Interestingly, a modified intraoperative imaging technique known as ICG–VAG in negative has been introduced to improve the localization and confirmation of dural AVFs across multiple studies (Julian et al., 2013 [71], 2015 [72]; Koyanagi et al., 2021 [73]), particularly in cases where 2D DSA is negative or inconclusive. This approach involves temporarily occluding the vessel suspected of being pathological before administering ICG, and then observing changes in the vascular filling pattern before and after clip removal. A rapid filling of the venous network upon releasing the clip confirms the shunt’s dependence on the occluded vessel. The technique enhances intraoperative decision-making by providing a dynamic, reversible, and intuitive method to test the hemodynamic role of individual vessels. Compared to conventional ICG–VAG, which can suffer from overlapping arterial and venous phases, the “negative” approach offers clearer interpretation and may reduce the need for repeated injections or immediate postoperative 2D DSA.

It is also worth mentioning that Hayashi et al. (2015) [74] reported the intraoperative use of a portable fluoroscopy unit equipped with a simplified 2D DSA function during surgical treatment of SVMs. Despite lower image resolution compared to conventional 2D DSA systems, this approach allowed real-time confirmation of shunt occlusion and was supported by ICG–VAG. Their experience highlights the potential of cost-effective intraoperative imaging modalities for vascular lesion management, especially in institutions without access to hybrid operating rooms.

**Table 2:** Comparison of intraoperative and perioperative vascular imaging modalities for SVMs. MRI = Magnetic Resonance Imaging, MRA = Magnetic Resonance Angiography.

Modality	Use Case	Spatial/Temporal Resolution	Quantitative Potential	Radiation/Contrast	When to Choose
**2D DSA**	Intraoperative guidance and confirmation of feeder and venous drainage control.	High spatial/very high temporal resolution (ms scale).	High—dynamic flow and embolic progression assessment.	Radiation + iodinated contrast; arterial catheterization.	Gold standard for intraoperative decision-making, complex fistulas, embolization monitoring, deep lesions [4,75]
**3D RA**	Pre- or intraoperative 3D vascular mapping and navigation.	Very high spatial/limited temporal.	Moderate—excellent geometry, limited dynamic insight.	Radiation + iodinated contrast.	When detailed 3D vascular anatomy is required (e.g., multi-segment feeders, surgical planning, spinal level uncertainty) [76,77,78].
**CTA**	Preoperative anatomical survey and vessel localization.	High spatial/no temporal resolution.	Low—structural information only.	Radiation + iodinated contrast.	When MRI/MRA is unavailable/contraindicated or as rapid whole-spine vascular overview with bone context [79,80,81].
**ICG–VAG**	Intraoperative real-time visualization of exposed vessels.	High spatial/high temporal (seconds).	Low—qualitative surface flow.	No radiation; i.v. ICG.	When direct visualization of intraoperative arterialization/venous drainage is needed; superficial fistulas [52,53,54].
**MRA**	Non-invasive assessment and localization of suspected SVM; evaluation of venous congestion and cord signal abnormalities.	High spatial/moderate-to-high temporal depending on sequence (seconds).	Moderate—dynamic enhancement, time-of-arrival mapping in advanced protocols.	No radiation; gadolinium contrast may be used depending on the sequence.	When screening for SVMs, for follow-up, or when CTA/2D DSA is inconclusive, first-line non-invasive imaging in suspected venous congestive myelopathy [80,81,82,83].

Finally, intraoperative use of Computed Tomography Angiography (CTA) and 3D Rotational Angiography (3D RA) was reported only once each. CTA was used during surgery in the case reported by Morris et al. (2011) [84] to confirm that the arterialized vein was located on the dorsum of the cord. Similarly, Ikezawa et al. (2021) [85] described a case using intraoperative 3D RA to precisely guide direct vertebral artery puncture and embolization in a hybrid operating room setting.

## 6. Post-Treatment and Follow-Up Role of Angiography

The role of 2D DSA for postoperative evaluation and follow-up of SVMs has been recognized for decades, with early case reports describing its use as early as 1987 [86] and 1992 [87]. Over time, 2D DSA has become a common tool to verify technical success after endovascular or microsurgical treatment, as consistently reported in numerous case series and reports published in the last years [88,89,90,91,92].

Although it can be considered the current reference standard for immediate post-embolization evaluation, 2D DSA is inherently invasive, involving arterial catheterization, iodinated contrast administration, and radiation exposure. As such, non-invasive techniques could be preferred for follow-up purposes, particularly in patients with stable outcomes or when long-term monitoring is needed. Magnetic resonance angiography (MRA) may be selected not only for its favorable safety profile, but also for its ability to simultaneously visualize vascular anatomy, osseous structures, and spinal cord signal alterations [93,94,95,96]. Time-of-flight MRA (TOF–MRA), a non-contrast, flow-dependent technique, demonstrates high sensitivity and specificity relative to 2D DSA [97], but its dependence on inflow effects limits performance in low-flow fistulas and increases the risk of false-positive flow-related hyperintensities [98]. Contrast-enhanced MRA (CE–MRA) improves vessel conspicuity yet may show lower sensitivity than TOF–MRA for detecting residual aneurysms due to contrast-bolus timing challenges and a narrow acquisition window [97]. Time-resolved CE–MRA sequences (CE–trMRA), including TRICKS (Time-Resolved Imaging of Contrast Kinetics), which share low-frequency k-space data across time frames, and TWIST (Time-Resolved Angiography With Interleaved Stochastic Trajectories), which uses variable-density k-space sampling to enhance both spatial and temporal resolution, have improved the dynamic assessment of spinal vascular lesions and show strong concordance with 2D DSA [99,100,101]. More recently, radial-based CE–trMRA has demonstrated improved robustness to motion and undersampling artifacts—a key advantage in spinal imaging, where swallowing, respiratory motion, and cerebrospinal fluid pulsation may impair diagnostic quality. Radial sampling mitigates streaking and ghosting [102] and improves stability in dynamic acquisitions. While specific spinal follow-up studies using radial CE–trMRA are not yet available to the best of our knowledge, aortic data support enhanced motion tolerance and diagnostic reliability with compressed-sensing radial acquisitions [103], highlighting their potential applicability to spinal vascular imaging.

Similarly, CTA is non-invasive and can show the surrounding anatomical structure [104,105]. ICG–VAG has also been proposed as a complementary or even substitute tool for postoperative 2D DSA in select cases, allowing confirmation of complete disconnection of the fistulous points [104,106,107,108,109].

Generally, the choice of imaging modality for post-treatment assessment and follow-up depends on multiple factors, including the type and complexity of the SVM, the technical approach used (microsurgical or endovascular), procedural outcomes, and patient-specific considerations.

Regarding follow-up intervals, most reports focus on immediate intraoperative or early postoperative imaging, with relatively limited data on structured long-term surveillance. 2D DSA has been employed for follow-up at 4, 10, and 12 months post-treatment in some studies [63,89,110], whereas a few others describe longer-term imaging at 5, 8, and 10 years [93,111,112], often combining MRA and 2D DSA for comprehensive evaluation. The non-invasive alternatives could play a main role in long-term monitoring, because of their safer nature, but also because most of the long-term monitored cases involved special cases, especially for complex cases where understanding the surrounding anatomy is crucial and cannot be fully addressed with 2D DSA alone.

## 7. Future Directions in Angiographic Imaging

Several promising avenues are emerging for the future development of angiographic techniques for the treatment of SVMs. While 2D DSA and ICG–VAG remain the cornerstone techniques for intraoperative guidance and post-treatment assessment, ongoing research aims to expand their capabilities beyond qualitative visual interpretation. One area of focus is the extraction of quantitative imaging metrics to objectively evaluate treatment outcomes [113]. Angiographic-based approaches such as parametric mapping [114,115,116] and frame-by-frame time–density curve analysis have shown potential in other vascular pathologies for identifying residual arteriovenous connections or persistent shunt flow with abnormal arterial–venous transit times.

Another area of interest concerns the personalization of treatment through patient-specific hemodynamic models. Recent studies on cerebral and peripheral vascular malformations (VMs) have shown that combining 3D angiographic imaging with flow data enables the construction of computational models useful for simulating and optimizing therapeutic approaches [117,118,119,120]. However, these techniques have not yet been explored in the context of SVMs.

The application of artificial intelligence (AI) to angiographic imaging represents another underexplored opportunity. While AI-driven analysis has been successfully implemented in cerebral AVMs to automate nidus segmentation and angiographically characterize the connected vessels [121,122], predict rupture risk [123], and automatically classify using CE–trMRA and 2D DSA data [114,115,124,125,126], similar applications are not yet available for spinal lesions. Extending the application of machine learning and AI algorithms to SVMs imaging could represent a promising future direction to address current treatment challenges, which may reduce operator dependence in the future.

Finally, building consensus guidelines that incorporate these newer approaches will be essential to ensure consistent, evidence-based, and patient-centered care in the management of SVMs.

## 8. Conclusions

The management of SVMs has evolved toward increasingly precise, physiology-oriented strategies that integrate intraoperative flow control, selective imaging, and structured follow-up. In this review, key elements of treatment planning and perioperative assessment are summarized in Table 1 and Table 2, providing a concise framework for surgical decision-making and postoperative evaluation.

2D DSA remains the reference standard for intraoperative guidance and post-treatment assessment in SVMs, owing to its unmatched real-time and high-resolution visualization of vascular anatomy and flow dynamics. Intraoperative ICG–VAG, while more limited in field of view, has emerged as a valuable adjunct that enhances surgical decision-making, facilitates preservation of normal venous structures, and enables immediate confirmation of fistula disconnection. Together, these techniques provide a multimodal foundation for optimizing intraoperative safety and efficacy.

Nevertheless, the invasiveness of 2D DSA, the qualitative nature of ICG–VAG, and anatomical constraints limit their use as stand-alone modalities, particularly in long-term follow-ups and in anatomically complex lesions. Future developments in quantitative image analysis, fluorescence-based refinements, advanced computational modeling, and AI-driven approaches have the potential to transform angiography from a descriptive tool into a platform for precision planning and monitoring. Establishing standardized multimodal protocols and consensus guidelines will be crucial to ensure evidence-based, reproducible, and patient-centered care. Ultimately, the evolution of angiographic technology should aim not only to improve procedural safety and efficacy but also to advance long-term outcomes and quality of life for patients affected by these rare and complex lesions.

## Data Availability

No new data were created or analyzed in this study. Data sharing is not applicable to this article.

## Abbreviations

The following abbreviations are used in this manuscript:

2D DSA	Two-Dimensional Digital Subtraction Angiography
3D RA	Three-Dimensional Rotational Angiography
AI	Artificial Intelligence
AKA	Artery of Adamkiewicz
ASA	Anterior Spinal Artery
AVF	Arteriovenous Fistula
AVM	Arteriovenous Malformation
CE–MRA	Contrast-Enhanced Magnetic Resonance Angiography
CE–trMRA	Contrast-Enhanced time resolved Magnetic Resonance Angiography
CTA	Computed Tomography Angiography
EVT	Endovascular Treatment
FL–VAG	Fluorescein Videoangiography
i.a.	Intra-arterial
ICG	Indocyanine Green
ICG–VAG	Indocyanine Green Videoangiography
i.v.	Intravenous
IONM	Intraoperative Neurophysiological Monitoring
MRA	Magnetic Resonance Angiography
MRI	Magnetic Resonance Imaging
NIR	Near-Infrared
PSA	Posterior Spinal Artery
SVM	Spinal Vascular Malformation
TOF–MRA	Time of Flight Magnetic Resonance Angiography
VM	Vascular Malformation

## References

[1] Schuette A.J., Cawley C.M., Barrow D.L. (2010). Indocyanine Green Videoangiography in the Management of Dural Arteriovenous Fistulae. Neurosurgery.

[2] Flores B.C., Klinger D.R., White J.A., Batjer H.H. (2017). Spinal vascular malformations: Treatment strategies and outcome. Neurosurg. Rev..

[3] Ho W.M., Gorke A., Petr O., Thome C. (2018). Treatment strategies of spinal arteriovenous fistulas and malformations: Timing matters. J. Neurosurg. Sci..

[4] Eddleman C.S., Jeong H., Cashen T.A., Walker M., Bendok B.R., Batjer H.H., Carroll T.J. (2009). Advanced noninvasive imaging of spinal vascular malformations. Neurosurg. Focus.

[5] Bemporad J.A., Sze G.S. (2000). MR imaging of spinal cord vascular malformations with an emphasis on the cervical spine. Magn. Reson. Imaging Clin. N. Am..

[6] Gailloud P. (2021). Spinal vascular malformations: Angiographic evaluation and endovascular management. Handb. Clin. Neurol..

[7] Talenti G., Vitale G., Cester G., Della Puppa A., Faggin R., Causin F. (2017). Rare association between spinal dural arteriovenous fistulas and dysraphisms: Report of two cases and review of the literature with a focus on pitfalls in diagnosis and treatment. Interv. Neuroradiol..

[8] Zyck S., Davidson C.L., Sampath R. (2025). Arteriovenous Malformations of the Central Nervous System. https://www.ncbi.nlm.nih.gov/books/NBK531479/.

[9] da Costa L., Dehdashti A.R., terBrugge K.G. (2009). Spinal cord vascular shunts: Spinal cord vascular malformations and dural arteriovenous fistulas. Neurosurg. Focus.

[10] Takai K. (2017). Spinal Arteriovenous Shunts: Angioarchitecture and Historical Changes in Classification. Neurol. Med. Chir..

[11] Krings T., Mull M., Gilsbach J.M., Thron A. (2005). Spinal vascular malformations. Eur. Radiol..

[12] Udelhoven A., Kettner M., Reith W. (2022). Spinal arteriovenous malformations. Radiologie.

[13] Andres R.H., Barth A., Guzman R., Remonda L., El-Koussy M., Seiler R.W., Widmer H.R., Schroth G. (2008). Endovascular and surgical treatment of spinal dural arteriovenous fistulas. Neuroradiology.

[14] Kiyosue H., Tanoue S., Okahara M., Hori Y., Kashiwagi J., Mori H. (2013). Spinal ventral epidural arteriovenous fistulas of the lumbar spine: Angioarchitecture and endovascular treatment. Neuroradiology.

[15] Castillo M. (2013). Digital Subtraction Angiography (DSA): Basic Principles. Vascular Imaging of the Central Nervous System: Physical Principles, Clinical Applications, and Emerging Techniques.

[16] Crone-Münzebrock W., Baake S., Thoma G., Müller P., Rehder U. (1985). Comparison of computed tomography and digital subtraction angiography for preoperative evaluation of soft-tissue tumors of the limbs. Arch. Orthop. Trauma. Surg..

[17] Dardik H., Miller N., Adler J., Ganti S.R., Myers D., Greweldinger J., Ibrahim I.M., Sussman B., Kahn M. (1986). Primary and adjunctive intra-arterial digital subtraction arteriography of the lower extremities. J. Vasc. Surg..

[18] Hertzer N.R., Flanagan R.A., O’Hara P.J., Beven E.G. (1986). Surgical Versus Nonoperative Treatment of Symptomatic Carotid Stenosis 211 Patients Documented by Intravenous Angiography. Ann. Surg..

[19] Pooley R.A., McKinney J.M., Miller D.A. (2001). The AAPM/RSNA Physics Tutorial for Residents: Digital Fluoroscopy. RadioGraphics.

[20] Crummy A.B., Turski P.A., Strother C.M. (2018). The History of Digital Subtraction Angiography. J. Vasc. Interv. Radiol..

[21] Manji F., Wang J., Norman G., Wang Z., Koff D. (2016). Comparison of dual energy subtraction chest radiography and traditional chest X-rays in the detection of pulmonary nodules. Quant. Imaging Med. Surg..

[22] Yeh B.M., FitzGerald P.F., Edic P.M., Lambert J.W., Colborn R.E., Marino M.E., Evans P.M., Roberts J.C., Wang Z.J., Wong M.J. (2017). Opportunities for new CT contrast agents to maximize the diagnostic potential of emerging spectral CT technologies. Adv. Drug Deliv. Rev..

[23] Guthaner D.F., Brody W.R., Lewis B.D., Keyes G.S., Belanger B.F. (1983). Clinical Application of Hybrid Subtraction Digital Angiography: Preliminary Results. Cardiovasc. Interv. Radiol..

[24] Brown P.A., Zomorodi A.R., Gonzalez L.F. (2017). Endovascular management of spinal dural arteriovenous fistulas. Arteriovenous and Cavernous Malformations.

[25] Gemmete J.J., Pandey A.S., Kasten S.J., Chaudhary N. (2013). Endovascular Methods for the Treatment of Vascular Anomalies. Neuroimaging Clin. N. Am..

[26] Pasternak J.J., Williamson E.E. (2012). Clinical Pharmacology, Uses, and Adverse Reactions of Iodinated Contrast Agents: A Primer for the Non-radiologist. Mayo Clin. Proc..

[27] Wei Y., Jiang X., Hibberd M., Sampedro A., Rautenbach J. (2025). Estimating the rate of acute adverse reactions to non-ionic low-osmolar contrast media: A systematic review and meta-analysis. Eur. Radiol..

[28] Spampinato M.V., Abid A., Matheus M.G. (2017). Current Radiographic Iodinated Contrast Agents. Magn. Reson. Imaging Clin. N. Am..

[29] Kim K.R. (2024). Percutaneous Sclerotherapy of Venous Malformations. Tech. Vasc. Interv. Radiol..

[30] Adler M., Mayo A., Zhou X., Franklin R.A., Meizlish M.L., Medzhitov R., Kallenberger S.M., Alon U. (2020). Principles of Cell Circuits for Tissue Repair and Fibrosis. iScience.

[31] Prasad R., Marotrao P.S., Sheorain V.S., Gamanagatti S. (2024). Safety and Efficacy of Lipiodol and N-Butyl Cyanoacrylate (N-BCA) Combination for Vascular Embolization. J. Clin. Interv. Radiol. ISVIR.

[32] Lopez O., Chevallier O., Guillen K., Comby P.O., Pellegrinelli J., Tinel C., Falvo N., Midulla M., Mourey E., Loffroy R. (2021). Selective Arterial Embolization with N-Butyl Cyanoacrylate Prior to CT-Guided Percutaneous Cryoablation of Kidney Malignancies: A Single-Center Experience. J. Clin. Med..

[33] Kojima T., Maeda T., Ito Y., Kikuta H., Fujii M. (2025). Onyx Liquid Embolic Agent: Basic Knowledge for Its Use in Interventional Neuroradiology. J. Neuroendovascular Ther..

[34] Vollherbst D.F., Chapot R., Bendszus M., Möhlenbruch M.A. (2022). Glue, Onyx, Squid or PHIL? Liquid Embolic Agents for the Embolization of Cerebral Arteriovenous Malformations and Dural Arteriovenous Fistulas. Clin. Neuroradiol..

[35] Ohlsson M., Consoli A., DiMaria F., Sgreccia A., Rodesch G. (2022). Natural history and management of spinal cord arteriovenous shunts in pregnancy: A monocentric series of 10 consecutive cases with emphasis on endovascular treatment. J. Neuroradiol..

[36] Opitz M., Zensen S., Bos D., Li Y., Styczen H., Wetter A., Guberina N., Jabbarli R., Sure U., Forsting M. (2021). Radiation exposure in the endovascular therapy of cranial and spinal dural arteriovenous fistula in the last decade: A retrospective, single-center observational study. Neuroradiology.

[37] Amarouche M., Hart J., Siddiqui A., Hampton T., Walsh D. (2015). Time-Resolved Contrast-Enhanced MR Angiography of Spinal Vascular Malformations. Am. J. Neuroradiol..

[38] McGraw J.K. (2004). Interventional Radiology of the Spine: Image-Guided Pain Therapy.

[39] Gao S.J., Zhang M.W., Liu X.P., Zhu Y.S., Liu J.H., Wang Z.H., Zang P.Z., Shi Q., Wang Q., Liang C.S. (2009). The clinical application studies of CT spinal angiography with 64-detector row spiral CT in diagnosing spinal vascular malformations. Eur. J. Radiol..

[40] Boni L., David G., Mangano A., Dionigi G., Rausei S., Spampatti S., Cassinotti E., Fingerhut A. (2015). Clinical applications of indocyanine green (ICG) enhanced fluorescence in laparoscopic surgery. Surg. Endosc..

[41] Yannuzzi L.A. (2011). Indocyanine Green Angiography: A Perspective on Use in the Clinical Setting. Am. J. Ophthalmol..

[42] Flower R.W. (1973). Injection technique for indocyanine green and sodium fluorescein dye angiography of the eye. Investig. Ophthalmol..

[43] Flower R.W., Hochheimer B.F. (1976). Indocyanine green dye fluorescence and infrared absorption choroidal angiography performed simultaneously with fluorescein angiography. Johns Hopkins Med. J..

[44] Reinhart M.B., Huntington C.R., Blair L.J., Heniford B.T., Augenstein V.A. (2016). Indocyanine Green: Historical Context, Current Applications, and Future Considerations. Surg. Innov..

[45] Norat P., Soldozy S., Elsarrag M., Sokolowski J., Yaǧmurlu K., Park M.S., Tvrdik P., Kalani M.Y.S. (2019). Application of Indocyanine Green Videoangiography in Aneurysm Surgery: Evidence, Techniques, Practical Tips. Front. Surg..

[46] Alander J.T., Kaartinen I., Laakso A., Pätilä T., Spillmann T., Tuchin V.V., Venermo M., Välisuo P. (2012). A Review of Indocyanine Green Fluorescent Imaging in Surgery. Int. J. Biomed. Imaging.

[47] Caglar Y.S., Ozdemir M., Kahilogullari G., Bozkurt M., Attar A. (2018). Management of Spinal Arteriovenous Fistulae with Intraarterial Indocyanine Green Angiography: A Case Report. Turk. Neurosurg..

[48] Jung Y., Lindgren A., Ahmed S.U., Radovanovic I., Krings T., Andrade-Barazarte H. (2023). Intraoperative intraarterial indocyanine green video-angiography for disconnection of a perimedullary arteriovenous fistula: Illustrative case. J. Neurosurg. Case Lessons.

[49] Takamiya S., Yamazaki K., Tokairin K., Osanai T., Shindo T., Seki T., Fujimura M. (2021). Intraoperative Identification of the Shunt Point of Spinal Arteriovenous Malformations by a Selective Arterial Injection of Saline to Subtract Signals of Indocyanine Green. World Neurosurg..

[50] Klingler J.H., Gizaw C., Blaß B.I., Hohenhaus R., Neidert N., Neumann-Haefelin E., Kotsis F., Grauvogel J., Scheiwe C., Beck J. (2024). Intraoperative indocyanine green (ICG) videoangiography in spinal hemangioblastoma surgery: Helpful tool or unnecessary?. Clin. Neurol. Neurosurg..

[51] Koyalmantham V., Kale S.S., Devarajan L.J., Phalak M., Chandra P.S., Suri A., Kumar R., Tandon V. (2020). Patient Outcomes Following Obliteration of Spinal Dural Arteriovenous Fistula and the Role of Indocyanine Green Angiography Videoangiography (ICG-VA) During Surgery. Neurol. India.

[52] Foster C.H., Morone P.J., Tomlinson S.B., Cohen-Gadol A.A. (2019). Application of Indocyanine Green During Arteriovenous Malformation Surgery: Evidence, Techniques, and Practical Pearls. Front. Surg..

[53] Walsh D.C., Zebian B., Tolias C.M., Gullan R.W. (2014). Intraoperative indocyanine green video-angiography as an aid to the microsurgical treatment of spinal vascular malformations. Br. J. Neurosurg..

[54] Jing L., Su W., Guo Y., Sun Z., Wang J., Wang G. (2017). Microsurgical treatment and outcomes of spinal arteriovenous lesions: Learned from consecutive series of 105 lesions. J. Clin. Neurosci..

[55] Sun L., Ren J., Wang L., Li J., He C., Ye M., Li G., Zhang H. (2020). Preservation of Coexisting Normal Superior Petrosal Vein in the Microsurgical Treatment of Superior Petrosal Sinus Dural Arteriovenous Fistulas Assisted by Indocyanine Green Video Angiography. World Neurosurg..

[56] Raabe A., Fichtner J., Gralla J. (2017). Advanced intraoperative imaging: Gold standard in brain and spine surgery?. Clin. Transl. Neurosci..

[57] Thorsteinsdottir J., Siller S., Dorn F., Briegel J., Tonn J.C., Schichor C. (2019). Use of a New Indocyanine Green Pooling Technique for Improved Visualization of Spinal Dural AV Fistula: A Single-Center Case Series. World Neurosurg..

[58] Ng Y.P., King N.K., Wan K.R., Wang E., Ng I. (2013). Uses and limitations of indocyanine green videoangiography for flow analysis in arteriovenous malformation surgery. J. Clin. Neurosci..

[59] Fox S., Hnenny L., Ahmed U., Meguro K., Kelly M.E. (2017). Spinal dural arteriovenous fistula: A case series and review of imaging findings. Spinal Cord Ser. Cases.

[60] Takai K., Kurita H., Hara T., Kawai K., Taniguchi M. (2016). Influence of indocyanine green angiography on microsurgical treatment of spinal perimedullary arteriovenous fistulas. Neurosurg. Focus.

[61] Su I.C., terBrugge K.G., Willinsky R.A., Krings T. (2013). Factors determining the success of endovascular treatments among patients with spinal dural arteriovenous fistulas. Neuroradiology.

[62] Daou B., Atallah E., Al-Saiegh F., Alkhalili K., Tjoumakaris S., Rosenwasser R.H., Jabbour P. (2017). Spinal Glomus Arteriovenous Malformation Manifesting with a Subarachnoid Hemorrhage. World Neurosurg..

[63] Baba H., Kiyosue H., Ide S., Onishi K., Kubo T., Tokuyama K. (2022). Spinal intraosseous arteriovenous fistulas with perimedullary drainage associated with vertebral compression fracture: Illustrative case. J. Neurosurg. Case Lessons.

[64] Takai K., Taniguchi M. (2012). Comparative Analysis of Spinal Extradural Arteriovenous Fistulas with or Without Intradural Venous Drainage: A Systematic Literature Review. Neurosurg. Focus.

[65] Takei J., Tochigi S., Arai M., Tanaka T., Kajiwara I., Hatano K., Ichinose D., Sakamoto H., Hasegawa Y., Ishibashi T. (2018). Spinal Extradural Arteriovenous Fistula with Cowden Syndrome: A Case Report and Literature Review Regarding Pathogenesis and Therapeutic Strategy. NMC Case Rep. J..

[66] Ito A., Endo T., Inoue T., Endo H., Sato K., Tominaga T. (2017). Use of Indocyanine Green Fluorescence Endoscopy to Treat Concurrent Perimedullary and Dural Arteriovenous Fistulas in the Cervical Spine. World Neurosurg..

[67] Mansour A., Endo T., Inoue T., Sato K., Endo H., Fujimura M., Tominaga T. (2019). Clipping of an anterior spinal artery aneurysm using an endoscopic fluorescence imaging system for craniocervical junction epidural arteriovenous fistula: Technical note. J. Neurosurg. Spine.

[68] Fukuda N., Yagi T., Kanemaru K., Yoshioka H., Hashimoto K., Senbokuya N., Ogiwara M., Kinouchi H. (2020). Anterior Approach Combined with Endoscopic Fluorescence Video Angiography for a Cervical Perimedullary Arteriovenous Fistula. World Neurosurg..

[69] Horiuchi R., Kanemaru K., Yoshioka H., Hashimoto K., Murayama H., Yagi T., Ogiwara M., Kinouchi H. (2020). Endoscope-Integrated Fluorescence Video Angiography for the Surgery of Ventrally Located Perimedullary Arteriovenous Fistula at Craniocervical Junction. World Neurosurg..

[70] Yu J. (2024). Embolization of an epidural arteriovenous fistula of the sacral nerve root with a neural tube defect: A case report. Int. J. Surg. Case Rep..

[71] Simal Julián J.A., Miranda Lloret P., López González A., Evangelista Zamora R., Botella Asunción C. (2013). Indocyanine green videoangiography “in negative”: Definition and usefulness in spinal dural arteriovenous fistulae. Eur. Spine J..

[72] Simal Julián J.A., Miranda Lloret P., Sanromán Álvarez P., Pérez de San Román L., Beltrán Giner A., Botella Asunción C. (2015). Indocyanine Green Videoangiography in Negative: Spinal Dural Arteriovenous Fistula. Glob. Spine J..

[73] Koyanagi I., Chiba Y., Imamura H., Osanai T. (2021). Intradural lumbar radicular arteriovenous malformation mimicking perimedullary arteriovenous malformation of the conus medullaris: Illustrative case. J. Neurosurg. Case Lessons.

[74] Hayashi K., Horie N., Morofuji Y., Fukuda S., Yamaguchi S., Izumo T. (2015). Intraoperative Angiography Using Portable Fluoroscopy Unit in the Treatment of Vascular Malformation. Neurol. Med.-Chir..

[75] Da Ros V., Picchi E., Ferrazzoli V., Schirinzi T., Sabuzi F., Grillo P., Muto M., Garaci F., Muto M., Di Giuliano F. (2021). Spinal vascular lesions: Anatomy, imaging techniques and treatment. Eur. J. Radiol. Open.

[76] Ropper A.E., Lin N., Gross B.A., Zarzour H.K., Thiex C., Chi J.H. (2012). Rotational angiography for diagnosis and surgical planning in the management of spinal vascular lesions. Neurosurg. Focus.

[77] Ozpeynirci Y., Schmitz B., Schick M., Konig R. (2017). Role of Three-Dimensional Rotational Angiography in the Treatment of Spinal Dural Arteriovenous Fistulas. Cureus.

[78] Jiang L., Huang C.G., Liu P., Zhang H., Liu Z.J., Liu B. (2008). Three-dimensional rotational angiography for the treatment of spinal cord vascular malformations. Surg. Neurol..

[79] Hu X., Yuan Z., Liang K., Chen M., Zhang Z., Zheng H., Cheng G. (2024). Application of Spinal Subtraction and Bone Background Fusion CTA in the Accurate Diagnosis and Evaluation of Spinal Vascular Malformations. AJNR Am. J. Neuroradiol..

[80] Oda S., Utsunomiya D., Hirai T., Kai Y., Ohmori Y., Shigematsu Y., Iryo Y., Uetani H., Azuma M., Yamashita Y. (2014). Comparison of dynamic contrast-enhanced 3T MR and 64-row multidetector CT angiography for the localization of spinal dural arteriovenous fistulas. AJNR Am. J. Neuroradiol..

[81] Takai K., Endo T., Fujimoto S. (2024). Angiographic challenges of spinal dural and epidural arteriovenous fistulas: Report on 45 cases. Neuroradiology.

[82] Bowen B.C., Pattany P.M. (1998). MR angiography of the spine. Magn. Reson. Imaging Clin. N. Am..

[83] Peckham M.E., Hutchins T.A. (2019). Imaging of Vascular Disorders of the Spine. Radiol. Clin. N. Am..

[84] Morris J.M., Kaufmann T.J., Campeau N.G., Cloft H.J., Lanzino G. (2011). Volumetric myelographic magnetic resonance imaging to localize difficult-to-find spinal dural arteriovenous fistulas: Report of 3 cases. J. Neurosurg. Spine.

[85] Ikezawa M., Izumi T., Nishihori M., Nagashima Y., Nishimura Y., Tsukuda T., Kropp A.E., Goto S., Otsuka T., Kato N. (2021). Direct vertebral artery puncture during open surgery for the endovascular treatment of a recurrent vertebro-vertebral arteriovenous fistula. World Neurosurg..

[86] Han S.S., Love M.B., Simeone F.A. (1987). Diagnosis and treatment of a lumbar extradural arteriovenous malformation. Am. J. Neuroradiol..

[87] Wakai S., Inoh S., Iwanaga H., Nagai M., Sato T., Izumi J. (1992). Successful surgical obliteration of a huge intradural arteriovenous fistula of the spinal cord in a child. Child’s Nerv. Syst..

[88] Nascimento F.A., Kan P., Sharp L., Mandel J.J. (2018). Spinal dural arteriovenous fistula and concomitant intramedullary spinal lesion. Can. J. Neurol. Sci..

[89] Ito K., Ryu B., Shima S., Mochizuki T., Sato S., Inoue T., Niimi Y. (2023). Pure spinal intraosseous arteriovenous fistula: A case report. Neuroradiol. J..

[90] Chiang S., Pet D.B., Talbott J.F., LaHue S.C., Douglas V.C., Rosendale N. (2023). Spinal epidural arteriovenous fistula with nerve root enhancement mimicking myeloradiculitis: A case report. BMC Neurol..

[91] Albiña Palmarola P., Khanafer A., El Mekabaty A., Forsting M., Ganslandt O., Henkes H. (2024). A ruptured craniocervical junction perimedullary arteriovenous fistula successfully treated through flow diversion: A case report. Surg. Neurol. Int..

[92] Bishwas S., Islam M.S., Shiplu M.H., Rana M.S., Ashfaq M., Rashid M., Alam F. (2022). Arteriovenous Malformation of Conus Medullaris Fed by the Artery of Desproges-Gotteron. J. Neurosci. Rural Pract..

[93] Abdalla R.N., Shokuhfar T., Hurley M.C., Ansari S.A., Jahromi B.S., Potts M.B., Batjer H.H., Shaibani A. (2021). Metachronous spinal pial arteriovenous fistulas: Case report. J. Neurosurg. Spine SPI.

[94] Ayhan S., Palaoglu S., Geyik S., Saatci I., Onal M.B. (2015). Concomitant intramedullary arteriovenous malformation and a vertebral hemangioma of cervical spine discovered by a pathologic fracture during bicycle accident. Eur. Spine J..

[95] Mizuhashi S., Kominami S., Fukuda K. (2020). Successful balloon-assisted coil embolization for a diagnostically difficult case of spontaneous vertebrovertebral arteriovenous fistula. Surg. Neurol. Int..

[96] Ogbu I.I., Tzerakis N., Al-Shamary Z. (2021). Sudden-onset paraplegia in a 72-year-old male with a spinal dural arteriovenous fistula: Illustrative case. JNS Case Lessons.

[97] van Amerongen M.J., Boogaarts H.D., de Vries J., Verbeek A.L., Meijer F.J., Prokop M., Bartels R.H. (2014). MRA versus DSA for follow-up of coiled intracranial aneurysms: A meta-analysis. Am. J. Neuroradiol..

[98] Schmidt V.F., Masthoff M., Czihal M., Cucuruz B., Häberle B., Brill R., Wohlgemuth W.A., Wildgruber M. (2021). Imaging of peripheral vascular malformations—Current concepts and future perspectives. Mol. Cell. Pediatr..

[99] Lindenholz A., TerBrugge K., van Dijk J., Farb R. (2014). The accuracy and utility of contrast-enhanced MR angiography for localization of spinal dural arteriovenous fistulas: The Toronto experience. Eur. Radiol..

[100] Kannath S., Mandapalu S., Thomas B., Enakshy Rajan J., Kesavadas C. (2019). Comparative Analysis of Volumetric High-Resolution Heavily T2-Weighted MRI and Time-Resolved Contrast-Enhanced MRA in the Evaluation of Spinal Vascular Malformations. Am. J. Neuroradiol..

[101] Khalafallah A.M., Tigre J.Y., Rady N., Starke R.M., Saraf-Lavi E., Levi A.D. (2024). Evaluating the diagnostic accuracy of 3D contrast-enhanced magnetic resonance angiography versus digital subtraction angiography in spinal dural arteriovenous fistulas. Neurosurg. Focus.

[102] Block K.T., Chandarana H., Milla S., Bruno M., Mulholland T., Fatterpekar G., Hagiwara M., Grimm R., Geppert C., Kiefer B. (2014). Towards Routine Clinical Use of Radial Stack-of-Stars 3D Gradient-Echo Sequences for Reducing Motion Sensitivity. J. Korean Soc. Magn. Reson. Med..

[103] Calastra C., Kleban E., Helfenstein F., Haupt F., Peters A., Huber A., von Tengg-Kobligk H., Jung B. (2024). Dynamic contrast-enhanced MRA of the aorta using a Golden-angle RAdial Sparse Parallel (GRASP) sequence: Comparison with conventional time-resolved cartesian MRA (TWIST). Int. J. Cardiovasc. Imaging.

[104] Kim A.Y., Khil E.K., Choi I., Choi J.A. (2020). Spinal extradural arteriovenous fistula after lumbar epidural injection: CT angiographic diagnosis using 3D-volume rendering. Skelet. Radiol..

[105] Shimizu T., Nagoshi N., Akiyama T., Suzuki S., Nori S., Tsuji O., Okada E., Yagi M., Watanabe K., Nakamura M. (2021). Surgical resection of arteriovenous fistula at the cauda equina. Spinal Cord Ser. Cases.

[106] Kular S., Tse G., Budu A., Bacon A., Choudhari K., Nagaraja S. (2020). Transarterial CT angiography for surgical planning of spinal dural arteriovenous fistula. Br. J. Radiol..

[107] Abdelazim A., Hartman C., Hooten K., Cutler A., Blackburn S. (2016). Neurologic decline after spinal angiography for dural arteriovenous fistula and improvement with emergent surgical ligation. World Neurosurg..

[108] Paolini S., Severino R., Cardarelli G., Missori P., Bartolo M., Esposito V. (2019). Indocyanine Green Videoangiography in the Surgical Treatment of Spinal Dural Arterovenous Fistula: A Useful Application. World Neurosurg..

[109] Subramaniam S.M., Ishii K., Sheng C.J., Nakatomi H., Takai K., Saito N. (2019). Successful surgical strategy for ventral thoracic spinal perimedullary spinal arteriovenous fistulas: Case report. Surg. Neurol. Int..

[110] Zhao J., Esemen Y., Rane N., Nair R. (2021). Intracranial subarachnoid haemorrhage caused by cervical spinal dural arteriovenous fistulas: Case report. Front. Neurol..

[111] Meder J.F., Devaux B., Merland J.J., Frédy D. (1995). Spontaneous disappearance of a spinal dural arteriovenous fistula. AJNR. Am. J. Neuroradiol..

[112] Iampreechakul P., Chuntaroj S., Wattanasen Y., Hangsapruek S., Lertbutsayanukul P., Siriwimonmas S. (2023). Spontaneous regression of extradural high-flow vascular malformation in spinal arteriovenous metameric syndrome (SAMS): A unique case report. Surg. Neurol. Int..

[113] Ionita C.N., Garcia V.L., Bednarek D.R., Snyder K.V., Siddiqui A.H., Levy E.I., Rudin S., Molthen R.C., Weaver J.B. Effect of injection technique on temporal parametric imaging derived from digital subtraction angiography in patient specific phantoms. Proceedings of the Medical Imaging 2014: Biomedical Applications in Molecular, Structural, and Functional Imaging.

[114] Frey S., Haine A., Kammer R., von Tengg-Kobligk H., Obrist D., Baumgartner I. (2017). Hemodynamic Characterization of Peripheral Arterio-venous Malformations. Ann. Biomed. Eng..

[115] Calastra C.G., Bono M., Granada A.B., Tuleja A., Bernhard S.M., Diaz-Zuccarini V., Balabani S., Obrist D., von Tengg-Kobligk H., Jung B. (2025). Hemodynamic Characterization of Peripheral Arterio-Venous Malformations Using Rapid Contrast-Enhanced MR Imaging: An In Vitro and In Vivo Study. Ann. Biomed. Eng..

[116] Schubert T., Wu Y., Johnson K.M., Wieben O., Maksimovic J., Mistretta C., Turski P. (2016). Time-of-Arrival Parametric Maps and Virtual Bolus Images Derived From Contrast-Enhanced Time-Resolved Radial Magnetic Resonance Angiography Improve the Display of Brain Arteriovenous Malformation Vascular Anatomy. Investig. Radiol..

[117] Stahl J., McGuire L.S., Abou-Mrad T., Saalfeld S., Behme D., Alaraj A., Berg P. (2025). Feasibility Study for Multimodal Image-Based Assessment of Patient-Specific Intracranial Arteriovenous Malformation Hemodynamics. J. Clin. Med..

[118] Zhang B., Chen X., Qin W., Ge L., Zhang X., Ding G., Wang S. (2024). Enhancing cerebral arteriovenous malformation analysis: Development and application of patient-specific lumped parameter models based on 3D imaging data. Comput. Biol. Med..

[119] Ganjkhanlou M.R., Shahidian A., Shahmohammadi M.R. (2024). Hemodynamic Study of Cerebral Arteriovenous Malformation: Newtonian and Non-Newtonian Blood Flow. World Neurosurg..

[120] Franzetti G., Bonfanti M., Tanade C., Lim C.S., Tsui J., Hamilton G., Díaz-Zuccarini V., Balabani S. (2021). A Computational Framework for Pre-Interventional Planning of Peripheral Arteriovenous Malformations. Cardiovasc. Eng. Technol..

[121] Forkert N.D., Illies T., Goebell E., Fiehler J., Säring D., Handels H. (2013). Computer-aided nidus segmentation and angiographic characterization of arteriovenous malformations. Int. J. Comput. Assist. Radiol. Surg..

[122] Forkert N.D., Säring D., Handels H. (2010). Automatic Analysis of the Anatomy of Arteriovenous Malformations using 3D and 4D MRA Image Sequences. Proceedings of the 13th World Congress on Medical Informatics.

[123] Nico E., Hossa J., McGuire L.S., Alara A. (2023). Rupture-Risk Stratifying Patients with Cerebral Arteriovenous Malformations Using Quantitative Hemodynamic Flow Measurements. World Neurosurg..

[124] Illies T., Forkert N.D., Ries T., Regelsberger J., Fiehler J. (2013). Classification of Cerebral Arteriovenous Malformations and Intranidal Flow Patterns by Color-Encoded 4D-Hybrid-MRA. Am. J. Neuroradiol..

[125] Chang W., Loecher M.W., Wu Y., Niemann D.B., Ciske B., Aagaard-Kienitz B., Kecskemeti S., Johnson K.M., Wieben O., Mistretta C. (2012). Hemodynamic Changes in Patients with Arteriovenous Malformations Assessed Using High-Resolution 3D Radial Phase-Contrast MR Angiography. Am. J. Neuroradiol..

[126] von Tengg-Kobligk H., Frey S., Obrist D., Baumgartner I., Lee B., Baumgartner I., Loose D., Yakes W. (2019). Arteriographic assessment: Is it still the gold standard for diagnosis of arteriovenous malformations?. Vascular Malformations.

